# A Narrative Review of Ethical Issues in the Use of Artificial Intelligence Enabled Diagnostics for Diabetic Retinopathy

**DOI:** 10.1111/jep.14237

**Published:** 2024-11-11

**Authors:** Alexandra Crew, Claire Reidy, Helene‐Mari van der Westhuizen, Mackenzie Graham

**Affiliations:** ^1^ Department of Continuing Education University of Oxford Oxford UK; ^2^ Nuffield Department of Primary Care Health Sciences University of Oxford Oxford UK; ^3^ Wellcome Center for Ethics and Humanities University of Oxford Oxford UK

**Keywords:** artificial intelligence, clinical practice, diabetic retinopathy, diagnostics, ethics, patient data, patient decision‐making and consent

## Abstract

**Introduction:**

Diabetic retinopathy is one of the leading causes of avoidable blindness among adults globally, and screening programmes can enable early diagnosis and prevention of progression. Artificial intelligence (AI) diagnostic solutions have been developed to diagnose diabetic retinopathy. The aim of this review is to identify ethical concerns related to AI‐enabled diabetic retinopathy diagnostics and enable future research to explore these issues further.

**Methods:**

This is a narrative review that uses thematic analysis methods to develop key findings. We searched two databases, PubMed and Scopus, for papers focused on the intersection of AI, diagnostics, ethics, and diabetic retinopathy and conducted a citation search. Primary research articles published in English between 1 January 2013 and 14 June 2024 were included. From the 1878 papers that were screened, nine papers met inclusion and exclusion criteria and were selected for analysis.

**Results:**

We found that existing literature highlights ensuring patient data has appropriate protection and ownership, that bias in algorithm training data is minimised, informed patient decision‐making is encouraged, and negative consequences in the context of clinical practice are mitigated.

**Conclusions:**

While the technical developments in AI‐enabled diabetic retinopathy diagnostics receive the bulk of the research focus, we found that insufficient attention is paid to how this technology is accessed equitably in different settings and which safeguards are needed against exploitative practices. Such ethical issues merit additional exploration and practical problem‐solving through primary research. AI‐enabled diabetic retinopathy screening has the potential to enable screening at a scale that was previously not possible and could contribute to reducing preventable blindness. It will only achieve this if ethical issues are emphasised, understood, and addressed throughout the translation of this technology to clinical practice.

## 1 Introduction

Diabetic retinopathy is an eye condition caused by diabetes‐related damage to the retina and is one of the leading causes of avoidable adult blindness globally [1]. As the burden of diabetes increases globally, so does diabetic retinopathy, and without proactive screening, it is often challenging to diagnose until vision loss has occurred [1, 2]. If diabetic retinopathy is diagnosed early, improved blood glucose management and ophthalmologic treatments can slow progression and prevent blindness [3]. Screening guidelines in the United States and United Kingdom recommend that individuals living with diabetes are screened annually or biennially for diabetic retinopathy, which can lead to a large volume of screenings (e.g., 2+ million individuals each year in England) [4, 5].

Presently, many screening programmes are undertaken manually in which a trained health worker captures an image of an individual's eyes, which is then evaluated by the same or a different health professional with the required training [6]. The cost of skilled human labour involved in this process can be a barrier to scaling screening programmes [1, 3, 4, 7]. Additionally, delays in evaluating the images can lead to delayed communication of results, contributing to emotional distress and delays in treatment [7].

**FIGURE 1:**
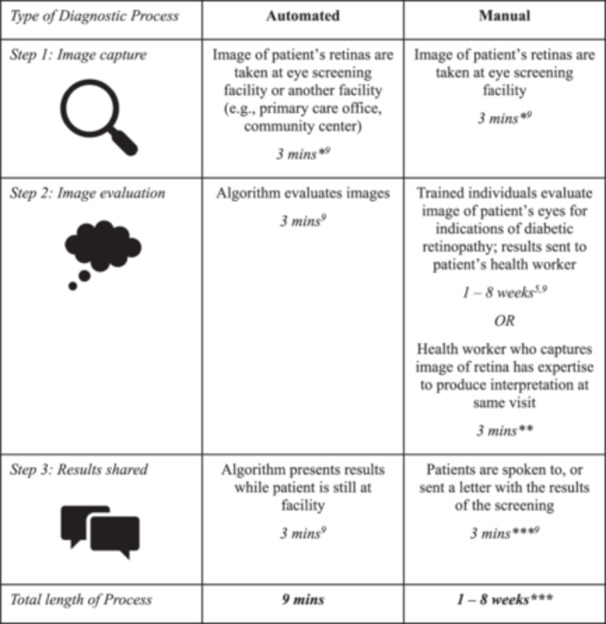
Example timelines for manual versus automated processes [5, 6, 9]. *Note:* *This does not include the overall time burden for the patient to access the facility or be seen. **This estimate is based on personal experiences from the author Helene‐Mari van der Westhuizen. ***The length of time between when the screening service or health worker receives the results and when these results are shared with the individual may vary (e.g., Health worker cannot reach patient for an additional 1 week, or lack of health worker/admin capacity to write to patient), leading to variation in the length of time of the manual diagnostic process as a whole.

These challenges have encouraged the development of artificial intelligence (AI) solutions for diagnosing diabetic retinopathy, along with the potential to reduce costs, scale screening programmes, and reduce wait times for results [1, 2, 4, 8]. Figure 1 illustrates the difference in timeline for diagnosing diabetic retinopathy using a manual versus automated process.

However, despite potential benefits, there are also risks to using these technologies. For example, if a certain group is underrepresented in the data on which an algorithm is trained (when an algorithm learns from data to generate an outcome), it might be more likely to make an incorrect diagnosis for someone who identifies as part of this less represented group [10, 11]. Further, given the sensitivity of patient data and AI's requirements to train on large amounts of data, this could also raise concerns regarding privacy protection for individuals whose data is used in training [12, 13].

Examining ethical issues related to AI can enable the prevention and minimisation of harm that might be associated with the use of this technology. Discussing and addressing these concerns can also facilitate benefits that appeal to developers of these technologies and related industry stakeholders. For example, protecting patient privacy could enhance users' confidence in the technology and as a result enable wider, safer adoption [4]. There is limited (albeit increasing) regulation of AI's use in healthcare, and illuminating ethical issues can help those in industry navigate this uncertainty [14]. Thus, the aim of this review is to identify ethical concerns related to AI‐enabled diabetic retinopathy and trigger further examination of these issues.

## 2 Methods

A narrative review was used to summarise, interpret, and critique a portion of the literature with the intent to deepen insight into the ethical considerations of AI‐enabled diabetic retinopathy diagnostics and consider this within the context of the broader body of work (AI ethics in healthcare and diagnostics) [15].

### 2.1 Search Strategy

We searched PubMed and Scopus to find papers about AI, diagnostics, ethics, and diabetic retinopathy, which yielded 1693 results (see Table 1 for search strings). We also reviewed the publications that cited the eight included papers from the database search and screening. This citation search yielded 185 papers, leading to a total of 1878 papers proceeding through the below screening process. The initial search was run in March 2023, and then it was updated and re‐run in June 2024.

**TABLE 1:** Databases and search string.

Databases	Search String
PubMed, Scopus	(artificial intelligence) OR (AI) OR (machine learning) OR (ML) OR (deep learning) OR (DL) OR (algorithm*) AND (diagnos*) OR (screen*) AND (ethic*) AND (diabetic retinopathy)

**FIGURE 2:**
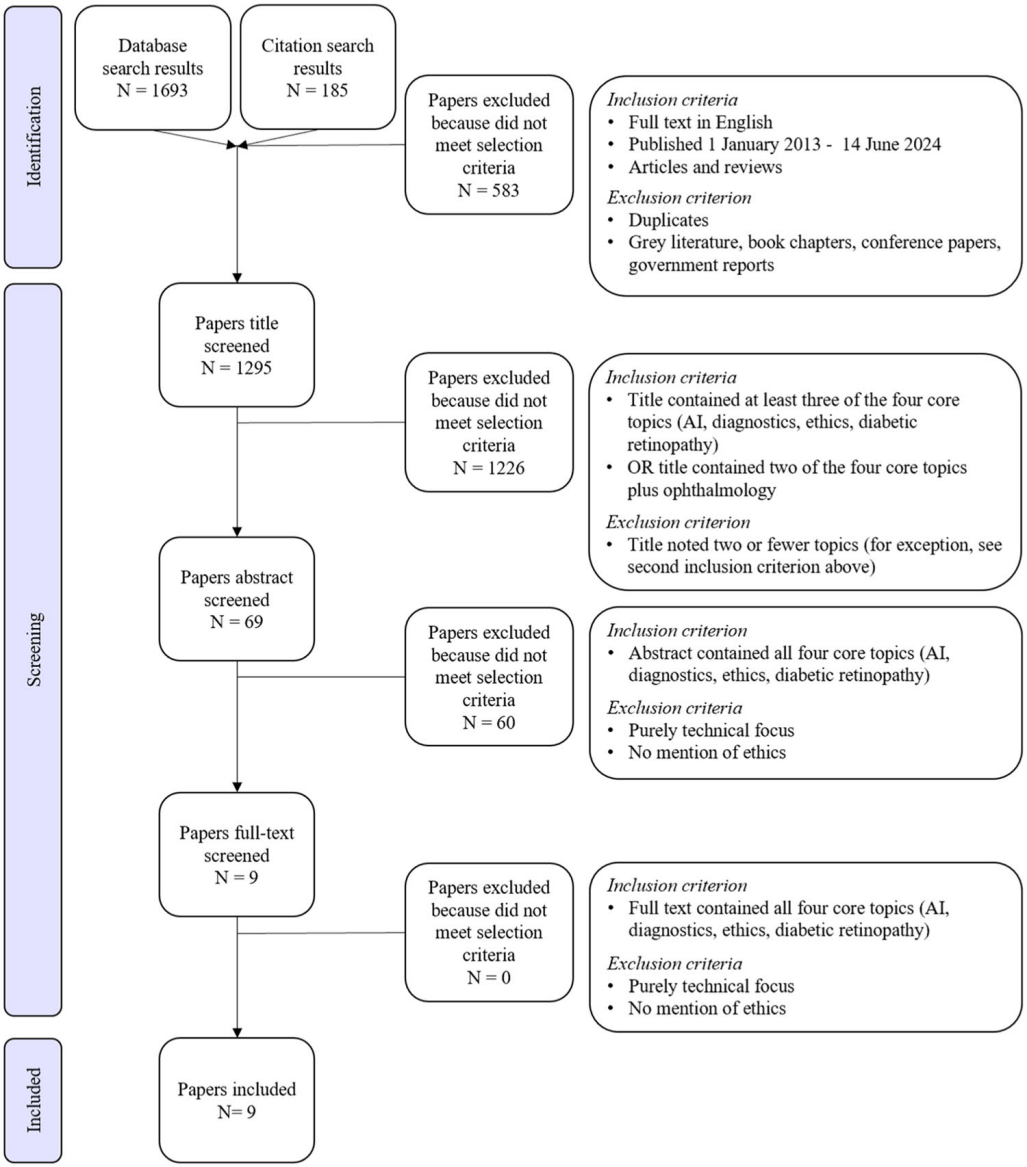
PRISMA flow chart.

We screened papers based on inclusion and exclusion criteria (outlined here in the PRISMA flow chart, Figure 2). We used gradually more stringent criteria to enable us to explore a wide range of papers at the top of the funnel of results, while ensuring we exclusively included papers closest to the intersection of key topics at the culmination of the screening process. For the first review stage, papers were filtered by the following inclusion criteria: full text in English, publication date between 1 January 2013 to 14 June 2024 (as the first AI‐enabled diabetic retinopathy diagnostic was approved in 2018), and primary research articles or peer‐reviewed evidence reviews [3]. Grey literature, book chapters, conference papers and reports were excluded. After the identification stage, 1295 papers progressed to title screening.

For the second review stage, we screened these titles based on whether they mentioned at least three of the four core topics OR two of the core topics plus ophthalmology. This enabled the inclusion of papers at or near the nexus of our four core concepts. After title screening, 69 papers progressed to abstract screening. Each paper's abstract was required to mention all four of the core topics. We used this to ensure each paper was relevant to our research at the intersection of the four core concepts. Subsequently, nine papers progressed to full text screening, which were all included in the review.

### 2.2 Data Analysis

Thematic analysis, as described by Braun and Clarke, was drawn on as an approach to analyse the selected papers [16]. Firstly, we familiarised ourselves with the data, reviewing the included papers line‐by‐line. AC then developed codes for where the authors of the included papers identified issues as (bio)ethical concerns, issues, challenges, and/or dilemmas related to the technology to create the coding framework. This coding framework was refined through further familiarisation with the data set and through discussion with co‐authors, drawing on their expertise in the ethics of AI. AC applied the coding framework to the data set and linked each data fragment with a code and summary of the data fragment using Microsoft Excel. We then sifted through the 21 codes and their related data fragments to identify patterns and grouped the ethical concerns into themes. We refined the themes to ensure they were cohesive and non‐duplicative until we settled on the themes shared in this paper. We also linked each data fragment with a translational stage (e.g., design, validation, implementation) and grouped them with at least one of two over‐arching stages (development and deployment) [17]. We explored the ethical issues in relation to translational stages because this is a relatively new technology with products moving along this translational continuum as they need integration into clinical practice.

## 3 Results

### 3.1 Overview of the Included Literature

Nine papers met the inclusion criteria for this narrative review, all of which were published between 2020 and 2023 (see Table 2 for papers included in the review). Seven out of nine papers are evidence reviews, one consists of original research conducted via a literature review and ethics content analysis, and another is a comparative study. Reviews that synthesised insight across the topics likely comprised the bulk of papers found in the search because this nexus of topics is niche and the field is early in development. The majority of papers were published in journals located in high‐income countries.

**TABLE 2:** Papers included in review.

Authors	Title	Journal	Year	Document type
Abdullah YI, Schuman JS, Shabsigh R, Caplan A, Al‐Aswad LA.	Ethics of artificial intelligence in medicine and ophthalmology	The asia‐pacific journal of ophthalmology	2021	Literature review
Abramoff MD, Tobey D, Char DS.	Lessons learned about autonomous AI: finding a safe, efficacious, and ethical path through the development process	American journal of ophthalmology	2020	Literature review
Grzybowski A, Brona P, Lim G, Ruamviboonsuk P, Tan GSW, Abramoff M, Ting DSW.	Artificial intelligence for diabetic retinopathy screening: A review	Eye	2020	Literature review
Grzybowski A, Singhanetr P, Nanegrungsunk O, Ruamviboonsuk P.	Artificial intelligence for diabetic retinopathy screening using colour retinal photographs: from development to deployment	Ophthalmology and therapy	2023	Literature review
Ng WY, Zhang S, Wang Z, Ong CJT, Gunasekeran DV, Lim GYS, Zheng F, Tan SCY, Tan GSW, Rim TH, Schmetterer L, Ting DSW.	Updates in deep learning research in ophthalmology	Clinical science	2021	Literature review
Raman R, Dasgupta D, Ramasamy K, George R, Mohan V, Ting D.	Using artificial intelligence for diabetic retinopathy screening: policy implications	Indian journal of ophthalmology	2021	Literature review
Sanamdikar ST, Patil SA, Patil DO, Borawake MP.	Enhanced detection of diabetic retinopathy using ensemble machine learning: A comparative study	Ingenierie des systemes d'Information	2023	Comparative study
Ursin F, Timmermann C, Orzechowski M, Steger F.	Diagnosing diabetic retinopathy with artificial intelligence: what information should be included to ensure ethical informed consent?	Frontiers in medicine	2021	Original research via literature review and ethics analysis
Zafar S, Mahjoub H, Mehta N, Domalpally A, Channa R.	Artificial intelligence algorithms in diabetic retinopathy screening	Current diabetes reports	2022	Literature review

### 3.2 Key Themes

We developed three key themes from the data which are presented in Table 3. Appendix A contains accompanying questions to guide further investigation of these ethical issues based on our analysis.

**TABLE 3:** Theme titles, descriptions, ethical issues, and associated translational stage(s).

Theme	Theme 1: Use of patient data	Theme 2: Patient consent and decision‐making	Theme 3: Clinical practice
*Description*	Ethical issues concerning the use and processing of patient data for activities related to this technology, such as training or diagnosis	Ethical issues related to patients' abilities to give informed consent [permission granted with understanding of the situation, including potential consequences [18]] and make decisions	Ethical issues related to the application of AI for diabetic retinopathy in clinical practice
*Ethical Issues*	Patient privacy Data ownership Exploitative practices Bias in training data	Patient consent for the use of their data Patient consent to the use of the technology in their care Transparency and explainability	Negative effects on the health worker‐patient relationship Professional replacement or displacement Dependence on the technology Responsibility, accountability, and liability Access to the technology
*Associated Translational Stage(s)*	Development and deployment	Development and deployment	Deployment
*Relevance to Diabetic Retinopathy*	The incidence of diabetic retinopathy is growing globally, which could lead to increased use of this technology and amplification of harm if patient privacy is violated. The global reach also makes representation in training data sets pertinent to ensure this technology can service individuals in varied contexts. The use of data in development and profits related to this technology make questions of data ownership and exploitative practices salient.	Considering the relative novelty of this technology, this may influence whether people feel comfortable with contributing their data to the AI‐enabled solutions. Making sure that consent is truly informed, with opportunities for questions and a balanced discussion of potential harms, is important.	Because this tool is used in a diagnostic process that historically involves a trained health worker and patient, and some versions of the technology can be used autonomously, its influence on this clinical context and health worker – patient interaction should be critically evaluated. Additionally, the global impact of diabetes, and the contributions of the social determinants of health to the incidence of type 2 diabetes, make access to novel technologies particularly relevant.

#### 3.2.1 Theme 1: Use of Patient Data

The literature describes ethical questions relating to the use of patient data with a focus on patient privacy, data ownership, and bias in training data. These could also intersect, for example, protecting patient data to prevent harm associated with compromising privacy occurs after the data has been obtained by the entity responsible for determining the purposes for which the data is used (e.g., a hospital, databank). Data ownership is important as it involves who is entitled to determine the circumstances in which it is shared or the purposes for which it is used, as well as who is entitled to profit from it (e.g., the data subject themselves, the data controller, the algorithm developer). Bias in training data is a concern once the data has been collated into a training data set, and one or more groups are under‐represented in the data set.

Privacy was identified as an ethical concern primarily because compromising it and inappropriately disclosing patient information can lead to a range of harms to data subjects, including discrimination related to insurance or employment, emotional distress, deterioration of trust, avoidance of care, and withholding important information from health workers [1, 13, 19, 20]. Because patient data is sensitive, used in training, validation, and diagnosis, insufficiently safeguarding it can have the negative consequences outlined above.

Data ownership was indicated as an ethical issue by Abdullah et al., identifying data ownership as a bioethical challenge, describing it as '*authority to control, process, or access data. It also conveys profitability from the right to sell data or to receive compensation. Medical data similar to physical property data ownership should be covered by property laws and/or intellectual right laws should decide ownership*'. [13] If patients' data are taken and used without appropriate compensation or if control is improperly revoked, this might be considered unethical. This is because taking, using, and benefiting from patients' data (e.g., a company profiting from an algorithm trained on patients' data) without appropriately compensating patients could be construed as taking unfair advantage of patients for the sake of one's own benefit. None of the papers included examples of how this compensation has been done in practice.

Multiple papers specifically focused on bias in training data sets [10, 13, 21]. Abdullah et al. noted that poor performance resulting from bias in training data can vary across groups and settings, which enables the uneven and potentially unfair distribution of benefits and risks associated with this technology [13]. Similarly, Ng et al. pointed out that bias in algorithm training data might lead to the algorithm underperforming for certain groups [10]. While the authors note that lack of representation of certain groups can lead to the algorithm offering incorrect or inconclusive results for these under‐represented groups, this differential performance could even lead to the algorithm being altogether unavailable to segments of the population if it cannot reliably offer accurate results. This uneven distribution of potential benefits and risks makes bias in training data sets a concern of justice.

#### 3.2.2 Theme 2: Patient Consent and Decision‐Making

This theme focuses on patients' abilities to make informed and autonomous decisions related to this technology, specifically patient consent for the use of one's data and consent to the use of the technology in one's care. The provision of information is central to enabling informed decisions.

If patients are not provided the opportunity to understand and make a decision regarding the use of their data and the use of the technology in their care, their autonomy could be threatened. Autonomy is facilitated by providing sufficient information to enable decision‐making [13, 21]. Abdullah et al. stated '*informed consent is based on the principle of autonomy. The informed consent could authorise partial or complete role of algorithms in health care services and detail the process of reaching diagnostic or therapeutic decisions by the machines. Clinicians should explain details of these processes to their patients*'. [13] Given the centrality of consent and decision‐making abilities to autonomy, lack of disclosure of the use of the patient's data or this tool along with the ability to consent or not might threaten patient autonomy.

Related to having sufficient information to enable decision‐making are transparency and explainability. Transparency is considered visibility around the data used and how this data were processed to arrive at the algorithm's output [13, 21]. Ursin et al. specifically included '*description of the AI's input and output data*' as information that should be provided to patients to enable informed consent [21]. Explainability is also important and refers to the AI tool providing explanations that can be perceived and understood by humans [10]. This relates to whether and how humans can understand the information provided and is distinct from transparency alone because explainability ensures the algorithm not only offers information but that it is comprehensible. As a result, a transparent and explainable algorithm is more likely to enable patient decision‐making and therefore, autonomy.

#### 3.2.3 Theme 3: Clinical Practice

Abdullah et al. indicated that negative effects on the health worker‐patient relationship should be considered, in addition to professional replacement or displacement (some or all of one's professional responsibilities are transferred to the technology), and dependence on technology. They state '*machine rivalry with doctors threatens to negatively impact doctor‐patient bonds or overtake jobs*'. [13] If a patient no longer sees their health worker because they receive their diagnosis from an autonomous diagnostic or if the entrance of this technology to health worker‐patient interactions influences the trust or respect the patient feels towards the health worker, this might negatively affect their relationship (e.g., the patient views the health worker as less capable because they perceive the health worker to be relying on a technology rather than their own expertise). As a consequence, the patient's fading trust in this health worker could influence their trust in health workers more broadly, leading to avoidance or delays in seeking care. Additionally, health workers' professional identity or self‐worth could erode [13]. Similarly, dependence on this technology could enable health workers to be misled by the tool, have deteriorating clinical skills over time, and experience negative psychological effects (e.g., deteriorated self‐confidence) [13].

Another component to clinical practice includes concerns around responsibility, accountability, and liability for medical errors [4, 13, 21]. Abdullah et al. suggested '*medical AI has many applications, all of which come with their concerns of malpractice and harm*'. [13] If a patient receives a false negative test result (e.g., showing they do not have diabetic retinopathy when they do) and resultantly does not receive treatment in a timely manner, their diabetic retinopathy could reach a point at which preventing or slowing the progression of blindness becomes difficult or impossible. This potential for harm raises questions regarding who should be responsible, accountable, and liable. These questions become particularly complex when the level of health worker and AI involvement can shift [13]. For example, if a diagnostic is approved for autonomous use, questions arise around whether the health worker who ordered the test is responsible for errors, or whether the responsibility lies with the developers of the algorithm, or elsewhere. Further questions arise if the algorithm is approved only for assistive use.

Ursin et al. noted infrastructure, for example dependence on an internet connection (which is common for many of the presently available AI‐enabled diabetic retinopathy diagnostics) and cost as potential influences on equitable access to this technology [21]. This unevenness in access to the technology can facilitate an uneven and potentially unfair distribution of the diagnostic's potential benefits. Rather than access to this technology being determined by need, the ability of a facility to use this technology could be influenced by internet access and funds, ultimately making differential access to the potential benefits of the technology in a clinical setting a concern of justice.

#### 3.2.4 Translational Stages

The first two themes were associated with both major translational stages, development and deployment, while the last theme was linked only to deployment, suggesting readers who wish to mitigate ethical concerns in the translation of this technology should keep in mind themes around the ‘Use of patient data’ along with ‘Patient consent and decision‐making’ through development and deployment while focusing on ‘Clinical practice’ in deployment.

## 4 Discussion

Our first key finding points to the importance of appropriate protection and ownership of patient data along with mitigating bias in training data. As the reach of diabetic retinopathy grows on a global scale, the potential for harm if privacy is violated expands and the importance of diagnostic performance across groups increases. Additionally, if use of the technology increases and profits grow along with it, questions of data ownership and compensation become more salient. Our second key finding, minimising obstacles to patient decision‐making (e.g., consent to use of their data, consent to the use of the technology in their care) suggests that guidance on the use of AI should include providing patients sufficient information, such as the scope of how their data will be used. Having transparent and explainable algorithms can enable this provision of information. Our third key finding was that attention should be paid to potential threats to the health worker‐patient relationship to minimise potential negative effects for both patients and health workers (including professional replacement, displacement, or dependence to mitigate potential negative effects). Responsibility, accountability, and liability must also be evaluated, particularly in the context of fluid levels of health worker involvement, as these can influence behaviour and how harms are addressed. Further, where health technologies have the potential to aggravate rather than ameliorate health inequities, access should remain a critical focus that is particularly relevant in this context due to the relationship between social determinants of health and type 2 diabetes [22]. Social determinants of health include factors such as socioeconomic status, food environment, and physical environment, and where individuals in vulnerable communities or resource‐constrained settings are at greater risk of type 2 diabetes and therefore diabetic retinopathy, the concern of access is further emphasised.

The themes surfaced in this review are nested within the ethical challenges that appear in broader discussions of AI ethics in healthcare and diagnostics [23, 24, 25, 26, 27, 28]. While this suggests the themes in this review are not unique to the context of diabetic retinopathy, it also underscores the importance of addressing these ethical problems across the healthcare ecosystem. Moreover, it might be possible to adapt strategies for addressing these issues deployed in other contexts to AI‐enabled diabetic retinopathy diagnostics. While versions of each of the ethical issues in these findings can be found in broader AI ethics literature, some issues surfaced in the broader literature were not emphasised in the papers we included, such as transformative effects (use of the technology influences unexpected re‐conceptualisations of reality) and cost‐effectiveness [23, 24].

Papers related to AI‐enabled diabetic retinopathy diagnostics often focus on technical performance (See e.g., Shah et al. (2021) [29] and Tan et al. (2019) [30]). By centering ethical issues particular to this topic and surfacing the themes of patient data, patient consent and decision‐making, and clinical practice, we hope to challenge the implicit prioritisation of technical considerations as these ethical concerns are just as important in the translation of this technology and ensuring harm is mitigated. Since over 100 million adults live with diabetic retinopathy and ~460 million individuals live with diabetes and therefore increased diabetic retinopathy risk globally (compared to a more narrow application with other diseases)—we highlighted justice (with regard to access and fair distributions of risks and benefits) as a consideration that is particularly important to this topic [31].

While this review provides useful insights on ethical concerns related to translating AI‐enabled diabetic retinopathy diagnostics, we also found two limitations in the literature: an under‐emphasis on the potentially exploitative practices related to data in a global context and insufficient exploration of equitable access. While data ownership was surfaced as an ethical issue, the discussion did not extend into considerations of the global regulatory and historical landscape. Because the use of patient information and medical devices are highly regulated in some countries, some companies might use their AI‐enabled diabetic retinopathy diagnostics in settings that have less stringent regulation to improve performance before applying for approval in settings with more rigid regulation. If a company were to collect data from individuals in a less regulated setting, use it to improve their technology, and then sell the improved technology for a profit exclusively in another setting, this might raise ethical questions regarding data ownership and exploitative practices. These questions can become particularly complex and nuanced when histories of exploitation are overlayed with how data might flow across regulatory landscapes without compensation.

Lastly, equitable access (particularly in a global context), which would form part of distributive justice, was under‐explored in the literature. While access was noted briefly as a concern of justice, this concern was not prominent across papers and explored in‐depth. Given diabetic retinopathy is growing globally and that social determinants of health (e.g., socioeconomic status, food environment, living location and environment) influence the development and management of type 2 diabetes, the equitable availability of this technology is an especially pertinent concern [22]. If this technology is dependent on infrastructure and demands prices that exclusively high‐resource settings can accommodate, then it could fail to enable scaling screenings in regions that could most benefit from the early identification of diabetic retinopathy.

Improving equitable access to diabetic retinopathy screenings is arguably one of the most important opportunities this technology can create. This technology has the potential to enable scaling screening programmes, reduce costs, and decrease wait times for results [1, 2, 4, 8]. These potential benefits can be particularly impactful in settings with low specialist to patient ratios, for example rural areas, and/or cost constraints because they can improve timeliness of results and increase access to diabetic retinopathy screenings for communities that might not otherwise receive these screenings [9, 32, 33, 34]. This can help prevent and slow diabetic retinopathy's progression towards blindness and ensure risk is not heightened among underserved populations [19]. This opportunity to improve equitable access can enable distributive justice and health justice more broadly, making it all the more critical to consider in the translation of these technologies.

Future research on data ownership and potential exploitation in a global context could provide valuable insight to inform legislation and regulation that mitigates risks of exploitation. Additionally, research on variability in this technology's availability across settings and influences on access and the distribution of potential benefits (e.g., price, product design, technology literacy) can shed light on how to improve availability across contexts (e.g., influence company incentives in an often profit‐driven health technology industry). Relatedly, future research should include and center perspectives from low‐resource settings, particularly in light of the opportunity for this technology to improve equitable access to diabetic retinopathy screenings in such contexts.

Future study designs that include primary data collection, instead of focusing on reviews, should include qualitative research with key stakeholders, such as users and those impacted by the presence or absence of the technology. This could advance this topic further by generating insights on how to address the ethical challenges raised in this review along with the effects on translation of addressing them. This information could identify potential solutions along with resultant advantages beyond being morally responsible that could incentivize developers to address these ethical concerns. Some examples include the potential for an algorithm's transparency to enable wider, safer adoption and for consistent performance across groups and settings to broaden the tool's total addressable market. In a similar vein, exploring if, how, and why ethical issues shift based on contextual factors (e.g., local regulation, economic setting, staff bandwidth) could enable developing and tailoring solutions to each setting.

Our review has several limitations. The small number of relevant and included papers is likely due to the intersection of topics (AI, diagnostics, ethics, and diabetic retinopathy) being niche and AI‐enabled diabetic retinopathy diagnostics becoming commercially available as recently as 2018. However, through mapping areas for future research, we hope to provide guidance on what should be investigated further. Additionally, we did not include conference proceedings in our search. This is because in many instances, conference proceedings do not include sufficient detail for comprehensive analysis, and instead, we focused on reviews and primary research papers that contain in‐depth datasets [35].

Another limitation is the prominence of authors on the included papers with company‐ and patent‐related interests as three of the nine papers fit this description [10, 12, 20]. These authors' ties might incentivize them to avoid revealing the full risks of such products (albeit this proximity also lends relevant expertise). This highlights the need for future, independent research.

## 5 Conclusion

We found that it is important for the use of AI in diabetic retinopathy screening to ensure patient data has appropriate protection and ownership and that bias be mitigated in training data. Barriers to patient decision‐making must be reduced through sufficient provision of information, with opportunity for discussion. Minimising potential negative effects in the context of clinical practice requires tending to the health worker‐patient relationship, professional displacement, replacement, and dependence, and responsibility, accountability, and liability. Because health workers have historically been involved in this diagnostic process, and in some cases will continue to be, we must understand and minimise these issues to avoid harm to patients or health workers. Access to this technology is also a particularly salient concern given the connection between social determinants of health and type 2 diabetes, the growing global burden of diabetic retinopathy, and the opportunity for this technology to improve equitable access to screenings. Health technologies often require infrastructure and prices that are more attainable in high‐resource settings, threatening to grow rather than close the digital divide. While this technology could enable scaling screenings, reducing preventable blindness, and enabling distributive justice, it will only be able to do so if we grant ethical issues salience throughout translation.

## Conflicts of Interest

The authors declare no conflicts of interest.

## Data Availability

Data sharing is not applicable to this article as no new data were created or analysed in this study.

## Appendix A

The below questions are intended to guide further exploration of the included ethical issues.

*Theme 1: Use of Patient Data*

Associated translational stage(s): Development and deployment

**Table:**

Issue	Considerations
Patient privacy	What are potential threats to patient privacy? Who has access to patient data? With whom is it appropriate to share this data? From whom should this data be protected? Who is responsible for this safeguarding? How might we protect patient data to ensure privacy? How might we balance this protection with sufficient flexibility to allow for innovation and potential benefits from data sharing (e.g., research that enables benefits for future patients)?
Data ownership	Who owns data used to train the algorithm? Who should own this data? What is owed to the people who own this data and how does this vary based on the type of data and its use? How is ownership and what is owed to owners determined?
Exploitative practices	Even if the individuals whose data were used to train this algorithm do not own their data, is there anything that is owed to them (e.g., access to the technology, shared profits)? Will these individuals have the opportunity to experience benefits the algorithm might enable in the future (e.g., shortened time‐to‐diagnosis enabled by the technology)?
Bias in training data	Have we audited the training data for bias? Is it representative of the population for which this diagnostic will be used? Are minorities sufficiently represented? Has the diagnostic been tested and validated with a diverse set of patients and users? Has it been tested and validated with the groups for which it will be used? Has it been tested and validated in varied real‐world settings (e.g., variable levels of lighting for taking photos of the patients' eyes) and/or the real‐world setting in which it will be used? Do the evaluation metrics for this tool include metrics to indicate performance across different groups? Will performance across groups be monitored on an ongoing basis?

*Theme 2: Patient Consent and Decision‐Making*

Associated translational stage(s): Development and deployment

**Table:**

Issue	Considerations
Patient consent for the use of one's data	Have patients whose data are being used in this algorithm, for example for training, consented to the use of their data? If so, how clear and visible was their ability to opt‐out? Are they aware of the full extent to which their data will be used? Do they have the opportunity to continually grant or revoke their consent?
Patient consent to the use of the technology in one's care	Is the use of this technology in care disclosed to patients? Do they have the option to decide if they would like to consent to the use of this technology? What information is needed for patients to provide informed consent for the use of one's data and the use of this technology in one's care? What is the best method for providing this information (e.g., videos, health worker communicates with the patient)? How can we ensure patients are given sufficient options and visibility of options to enable consent and/or the option to not consent (e.g., using an opt‐out model, co‐designing the consent process with patients)? Who is responsible for providing the patient with sufficient information and the opportunity to consent or not? Are there additional ways in which this tool might influence the autonomy of patients? How might we enable autonomy for patients (e.g., codesign for sufficient explainability to enable decision‐making for patients)?
Transparency and explainability	How transparent and explainable is this diagnostic? Can its input, processing of data, and output be understood by users? Is it sufficiently comprehensible for patients to make informed decisions, particularly regarding consent? Is it sufficiently comprehensible to audit the tool? How are we determining if it is sufficiently comprehensible? Are we consulting relevant users and stakeholders, including patients and health workers? How might we reasonably make this tool more explainable while balancing/maintaining accuracy?

*Theme 3: Clinical Practice*

Associated translational stage(s): Deployment

**Table:**

Issue	Considerations
Negative effects on the health worker‐patient relationship	How might this tool influence the health worker‐patient relationship? Could it influence trust in this relationship? How might this change with different levels of health worker involvement in the diagnostic process (e.g., if the tool is used autonomously or assistively)? Have we consulted health workers and patients as to the influence it has now or might have in the future on how they relate to each other? How might we mitigate any potential risks to the health worker‐patient relationship (e.g., codesign the tool's user interface and the steps for its use with health workers and patients; for an autonomous diagnostic, there could be a chat feature for the patient to send follow‐up questions to their health worker; for assistive AI, the interface could be designed to facilitate health worker‐patient communication regarding the result)?
Professional replacement or displacement	How might the introduction of this technology influence health workers' professional stability? How can we minimise potential negative effects (e.g., the workplaces of health workers can offer avenues of support, such as affirmation of value, support groups, and educational programmes, policy makers can create policies that facilitate and provide funding for re‐skilling and for support in transitioning to new roles)?
Dependence on the technology	Is there a risk for health workers to become dependent on these tools? How can we minimise potential negative effects, such as loss of skill or decreased confidence (e.g., workplaces of health workers can offer avenues of support, such as affirmation of value, support groups, and educational programmes)? Is it possible for the introduction of this technology to influence health workers in additional ways (e.g., if health workers feel their clinical decision‐making is stifled)? Have we consulted relevant stakeholders to understand their perceptions of what these influences might be? How might we address them (e.g., can potentially codesign solutions with relevant stakeholders)?
Responsibility, accountability, and liability	Who is responsible and accountable for the algorithms' diagnoses? The health worker? The developer? The healthcare organisation? Does this change for different categories (e.g., autonomous v. assistive) and varied levels of health worker involvement? What mechanisms are in place to incentivize relevant parties to prevent and ameliorate harm to patients related to the technology? What reporting and monitoring mechanisms are in place to enable identifying instances of patient harm and holding the appropriate party(ies) accountable?
Access to the technology	Are there groups or regions that might have increased or decreased access to this tool? What is influencing these different levels of access? What role does price play in influencing access? How might we change or remove these barriers to increase access (e.g., For developers: if different levels of lighting across settings are a barrier, potentially (if possible) enable the algorithm to analyse images taken with different levels of lighting; if cost is a barrier, can potentially price tools on a sliding scale; For policy makers: if internet is a barrier, can potentially create policies to facilitate expanding internet access; For health systems: if technology literacy is a barrier, can potentially provide educational materials and programmes)? How might the use of this technology vary across environments (e.g., if the diagnostic is operating autonomously without human supervision, how might the referral scheduling process differ from a diagnostic in a facility with humans guiding the patient experience and a front desk to schedule the referral?)? Are we designing this tool to be used for a particular group or setting? How does this design influence the settings and groups in which this tool can be used? Have we considered the perspectives of various stakeholders and how needs might vary across groups and contexts? Do the perspectives and biases of these stakeholders differ from our own? Are there additional ways we can incorporate varied perspectives and mitigate the influence of our own biases?

## Appendix B

See Table B1.

**TABLE B1:** Search terms by core concept.

AI	Diagnostics	Ethics	Diabetic retinopathy
artificial intelligence	diagnos*	ethic*	diabetic retinopathy
machine learning	screen*		
deep learning			
AI			
ML			
DL			
algorithm*			
